# The Effect of Lateral Wedge Insole on Gait Variability Assessed Using Wearable Sensors in Patients with Medial Compartment Knee Osteoarthritis

**DOI:** 10.1155/2023/6172812

**Published:** 2023-01-16

**Authors:** Yosuke Ishii, Masakazu Ishikawa, Hiroshi Kurumadani, Toru Sunagawa, Shota Date, Makoto Takahashi, Yoshitaka Iwamoto, Nobuo Adachi

**Affiliations:** ^1^Department of Biomechanics, Graduate School of Biomedical and Health Sciences, Hiroshima University, Hiroshima, Japan; ^2^Department of Orthopaedic Surgery, Faculty of Medicine, Kagawa University, Kagawa, Takamatsu, Japan; ^3^Department of Analysis and Control of Upper Extremity Function, Graduate School of Biomedical & Health Sciences, Hiroshima University, Hiroshima, Japan; ^4^Department of Orthopedic Surgery, Graduate School of Biomedical & Health Sciences, Hiroshima University, Hiroshima, Japan

## Abstract

**Background:**

Lateral thrust seen in people with medial compartment knee osteoarthritis can cause dynamic knee instability and poor postural control during gait cycles. A lateral wedge insole can reduce the lateral thrust and may have a favorable effect on gait variability, which in turn may indicate gait instability improves. The aim of this study was to investigate the effect of lateral wedge insole on gait variability in knee osteoarthritis patients.

**Method:**

We involved 15 symptomatic knee osteoarthritis patients who were provided with lateral wedge insole and 13 healthy asymptomatic volunteers as the control group. The gait variability was evaluated as the coefficient of variation of stride, stance, and swing duration based on acceleration monitoring using a wearable sensor. The lateral thrust was estimated as the lateral acceleration peak on the shank sensor. These measurements were performed without lateral wedge insole (baseline), immediately with lateral wedge insole (T0) at the initial office visit and one month after intervention (T1).

**Result:**

Our data showed that the stance duration coefficient of variation and lateral thrust at T1 in the knee osteoarthritis group, were significantly decreased compared to the baseline values and these values were identical to those in the control group.

**Conclusion:**

The lateral wedge insole reduces dynamic knee instability and could improve gait variability in medial compartment knee osteoarthritis.

## 1. Introduction

Medial knee osteoarthritis (OA) is a common musculoskeletal disease affecting populations worldwide [1]. This pathology is associated with several problems such as knee pain, limited range of motion, poor sensorimotor, and varus deformity [2–4] and often leads to fall events due to functional disability, especially impaired walking ability [5, 6]. Thus, understanding ambulatory dysfunction and its management are important for preventing fall events due to knee OA in daily clinical practice.

Knee OA often presents the lack of postural stability with knee instability during routine activities [7, 8] and was correlated with balance function which can predict fall events [9]. Gait is the regular and repetitive movement of the lower limbs; however, pathological OA patients with a lack of postural control present irregular motion of their limbs involuntarily [10, 11]. Gait variability is an indicator that can quantify to postural stability and showed to be predict to faller based on lack of balance ability when compared with gait speed and functional status by a previous study [12]. Moreover, the high gait variability was reported in patients with knee OA with an impaired functional ability [13] and was correlated with a poor clinical score [14] and fear of falling [15]. Then, the gait variability may serve as a potential indicator to assess ambulatory function in knee OA.

The involved knee with OA has structural instability on the frontal plane and show that excessively varus motion cause poor postural stability [16]. The lateral thrust has been recognized as dynamic knee instability and forced to aggravation of varus motion during gait [17]. On the other hand, the patients with knee OA who underwent total knee arthroplasty showed a reduction of lateral thrust [18], improvement in balancing ability [19], and gait variability [20], postoperatively. According to the evidence, the lateral thrust could contribute to gait variability, and minimizing of it could be a clinical target for patients with knee OA.

Lateral wedge insole (LWI) is a common conservative intervention in clinical practice for medial knee OA in Japan [21]. LWI has previously demonstrated a favorable effect on the symptoms and biomechanical functioning of the affected knee joint [22–24]. Furthermore, recent studies have reported the positive effect of the LWI on the reduction of structural instability in the medial compartment of the knee [25, 26]. Especially, The LWI reduces lateral thrust by inhibiting dynamic varus movement during gait [22, 27, 28]. These previous studies show LWI may have a favorable effect on gait variability by inhibiting varus instability. However, the effect of LWI on gait variability on knee OA remains unclear.

The aim of this study was to investigate the effect of LWI on the gait variability in patients with knee OA. Our hypothesis was that LWI improves gait variability with both immediate and longitudinal effects in patients with knee OA.

## 2. Materials and Methods

### 2.1. Participants

All consenting patients who visited our knee clinic and could walk without support were included in the present study. We involved the 15 symptomatic patients with knee OA (mean age, 64.9 ± 9.6 years; female: 6) presenting with medial knee pain, with radiographs showing osteophytes and medial joint space narrowing. Patients were classified using the Kellgren and Lawrence (KL) grading system to evaluate disease severity [29]. The varus knee alignment was determined by the femorotibial angle (FTA). We also recruited 13 healthy asymptomatic volunteers for a control group (mean age, 65.0 ± 4.4 years; female: 7).

As per detailed inclusion criteria, they diagnosed primary knee OA with no history of trauma, present of medial joint space narrowing (KL > 1), and varus alignment (FTA > 174 degrees). On the other hand, those with a history of a central or peripheral neurologic disorder such as a cerebrovascular accident or diabetic peripheral neuropathy, severe dementia, Parkinson's disease, or surgery of the knee which could affect their gait performance, were excluded from the study.

This trial was approved by the ethics review board of our institution, and it was in accordance with the Declaration of Helsinki, 1964, and comparable ethical standards. Informed consent was obtained from each participant.

### 2.2. Lateral Wedge Insole

The insole had an elevation with a lateral thickness of 7 mm (approximately 7° inclination) (Lateral Wedge Plus®, Nakamura Brace, Japan). The insole was made of silicon rubber and fitted to the patient's foot size by a prosthetist and orthotist which has been experience over 20 years. LWI was placed under the sole and fixed using a flap cover. The participants were provided with LWI to ensure the best individual fit and subjective comfort. All participants were instructed to wear the insole as much as possible in their daily life including weight-bearing situation as their standing and walking with and without outdoors to ensure a considerable amount of correction time. A single examiner checked the average wearing time per day at follow-up for each participant based on their self-reporting (Table 1).

### 2.3. Assessments of Gait Variability and Form

**Figure 1:**
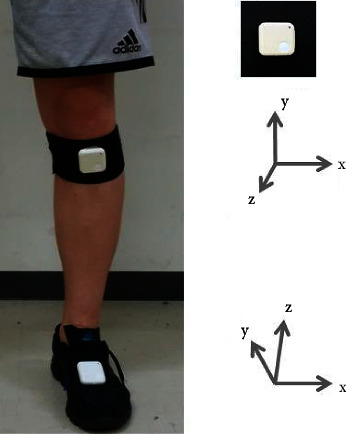
Joint axes for the foot and shank sensors.

In this study, we used two wearable sensors (WAA-010, ATR-Promotions, Japan) consisting of an acceleration sensor and a gyroscope with a sampling rate of 100 Hz. The sensors were placed on the dorsal foot and tibial tubercles using a tape and belt. The sensor placed on the shank was cautiously attached along the tibial longitudinally. Joint axes in the foot sensor were defined as follows: *y*-axis oriented in an anteroposterior direction; *x*- and *z*-axes in mediolateral and vertical directions. Contrastingly, in the shank sensor, the *y*-axis was oriented in a vertical direction, while the *x*- and *z*-axes were in mediolateral and anteroposterior directions (Figure 1).

The participants were asked to walk comfortably along a 10 m walkway, twice. Acceleration data during the middle four strides, in which the acceleration-waveform was visually stable and did not include the gait initiation and termination, were analyzed. To estimate the gait cycle, we used the vertical acceleration and sagittal angular velocity peaks on the foot and shank sensors. To detect the gait events, peak values were identified as heel-contact and foot-off in a single gait cycle based on a previous study [30]. A single stride was defined from one heel-contact to the next and the stance phase was from the heel-contact to the foot-off. The gait time for a 10 m walk was recorded with a stopwatch and gait speed was obtained. The stride length was calculated as the value of multiplication between gait speed, and stride duration which was obtained using the acceleration sensor. Furthermore, a single gait cycle was time-normalized to 101 data points based on the duration of a single stride. Regarding the additional information about gait abnormality, the lateral thrust was estimated as the lateral acceleration peak on the shank sensor up to the first 10% of the gait cycle, according to existing literature [22, 31]. All raw data were filtered to low-pass with a 10 Hz cut-off using MATLAB software (MATLAB 2015a, MathWorks, Japan). The gait variability was evaluated as the coefficient of variation (CV) of stride, stance, and swing duration based on four consecutive gait cycles.

### 2.4. Clinical Assessment

The clinical evaluation was based on a patient-subjective tool. Knee injury and osteoarthritis outcome score (KOOS) was a self-administered questionnaire and was constructed with several subscales [32]. We used the Japanese version which showed high valiability [33] and adopted all subscales for assessing including symptoms, pain, function, sports, and quality of life (QOL). A higher value on the KOOS implied better knee function.

In addition, a visual analogue scale (VAS) was used to evaluate knee pain [34], including the immediate and longitudinal reactions, when participants wore the LWI.

### 2.5. Follow-Up

In the knee OA group, a total of three follow-ups were performed, including the initial visit without LWI as the baseline, immediately with LWI (T0) at the initial office visit, and one-monthpost-intervention (T1). The KOOS evaluation was performed at baseline and T1. Contrary, the control group was measured once at baseline.

### 2.6. Statistical Analyses

All data were confirmed normality by the Shapiro–Wilk test. The demographic data were compared between groups using the unpaired *t*-test or Mann–Whitney *U* test. The KOOS were analyzed using paired *t*-test. Furthermore, the VAS, spatiotemporal gait parameters, lateral thrust, and CVs were analyzed within the knee OA group using repetitive one-way analysis of variance. To compare with the group, the nonrepetitive one-way analysis of variance was performed. The Bonferroni correction was performed as the post hoc test. All statistical analyses were performed using SPSS (v23, IBM, Japan), and the critical value was set at *p* < 0.05. Moreover, the CV data performed the post hoc test using G*∗*power and calculated the power based on the current sample size. When we investigated the effect of LWI on gait variability using repeated ANOVA, the effect size and statistical power were 0.48 and 0.99.

## 3. Results

### 3.1. The Demographic Data of the Participants

The demographic data of the participants are summarized in Table 1. The BMI among the knee OA group was significantly higher than that in the control group. No other significant difference was found in the demographic data between OA and control. All participants in the knee OA group continued wearing the LWI, and the average of duration time for LWI wearing was 8.1 ± 3.0 hours per day.

### 3.2. Lateral Thrust

**Figure 2:**
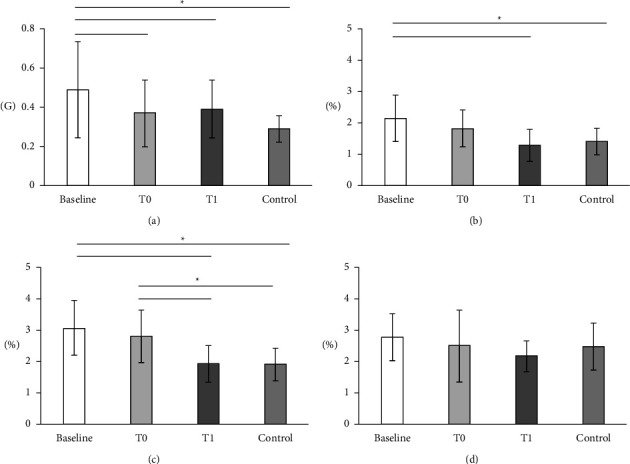
The comparison of the lateral thrust (a) and the coefficient of variation of stride (b), stance (c), and swing (d) duration. Values represent the mean ± standard. ^*∗*^denotes statistically significant difference between follow-up or group, *p* < 0.05.

In the Knee OA group, the peak values of the lateral acceleration at T0 and T1 were significantly lower when compared with that in baseline (Figure 2(a)).

Comparing between groups, at baseline, the peak value of the lateral acceleration in the knee OA group was significantly higher than that in the control group. However, there was no significant difference in the lateral thrust at T0 and T1 between OA and control groups (Figure 2(a)).

### 3.3. Spatiotemporal Gait Parameters and Gait Variability

The spatiotemporal gait parameters: gait speed, length, duration of stride, stance, and swing were not significantly different at follow-up for groups (Table 2).

In the knee OA group, the CVs of stride and stance duration at T1 were significantly lower than those at baseline. Moreover, the stance duration CV at T1 was significantly lower than that at T0 (Figures 2(b) and 2(c)). However, the swing duration CV was not different during the follow-up and between the groups (Figure 2(d)).

Comparing between groups, at baseline, the CVs of stride and stance duration were significantly higher than those in the control group, and the higher value of stance duration was remained at T0 (Figures 2(b) and 2(c)).

### 3.4. Subjective Clinical Evaluation

In the knee OA group, the values recorded using the VAS at T0 and T1 significantly decreased compared with that at baseline. All KOOS scores at T1 were significantly higher than the score at baseline (Table 3).

## 4. Discussion

This study investigated the effect of the LWI on gait variability based on wearable sensors in patients with knee OA. Our findings suggest that gait variabilities of stride and stance duration were longitudinally improved, but not immediate effect of the LWI.

The stance duration CV in the knee OA group after wearing the LWI for one month decreased, compared to that at baseline and T0. It was found that the value at T1 was not different from that of the control group. Thus, these findings indicate the longitudinal effect of the LWI use on gait variability, partly supporting our hypothesis. Most patients with knee OA had greater varus alignment and it was associated with postural instability during single-leg standing task [16]. The dynamic varus movement during the stance phase often presents as the lateral thrust, known as dynamic knee instability [17]. Previous studies suggest that lateral thrust can be reduced by the use of LWI; this effect was described by the acceleration sensor data [22, 27]. In this study, lateral thrust in the knee OA group was higher compared to the control group at baseline. However, following one month of wearing LWI, the lateral thrust reduced and was found to be similar in both the groups. Furthermore, the gait variability of the swing duration, which is not related to the lateral thrust, did not change. These findings could explain that the LWI improved the gait variability by supporting postural stability which inhibited the varus movement of the knee during the stance phase.

Our result did not show any immediate effect of the LWI on gait variability. Although lateral thrust and knee pain decreased at T0, gait variability did not change between baseline and T0. In contrast, previous biomechanical studies provided evidence that the LWI can immediately decrease the knee mechanical stress and pain [22, 23, 27, 28]. A possible explanation is that compensatory adjustments in associated joints following a sharp correction for the mechanical stress on the knee could potentially worsen compliance and gait variability. Khan et al. reported that wearing the LWI in combination with gait modification causes worsening of balance despite a sharp correction in knee mechanical stress, indicating the delicate trade-off between biomechanical correction and patient's comfort level [35]. Contrastingly, Esfandiari et al. reported improved balance following the use of LWI for four weeks [36]. Our results suggest that the gait variability did not change immediately because of the limited time for adaptation following the correction by the LWI. In other words, it might provide insight into the adaptive condition of insole.

Knee symptoms and pain are associated with sensorimotor function based on poor proprioception [37]; its changes might lead to the poor postural stability with the involved lower leg. Our results showed the VAS scores was consistently improved along with CV values at T1. Some previous studies about the effect of LWI [23, 38] reported the reduction of symptoms and pain, immediately and longitudinally due to controlling mechanical stress, and supported the natural reaction of LWI. Therefore, it raises concern that the effect of LWI on the gait variability might be affected by the improvement of clinical symptoms. However, our data showed that the VAS was improved at T0 although the CVs did not improve. Moreover, there was also no correlation between the amount of improvement of CV and VAS. Then, the longitudinal improvement of clinical scores could not contribute to the synergistic effects for gait variability because that its effect is small and not a directory.

Gait variability is strongly associated with poor balance, mobility, and greater fall events due to low physical function [39–41]. Therefore, high gait variability implies low ambulatory function and its detection and improvement could prevent fall events among the afflicted. The wearable sensor is a useful tool and provides valuable information about the spatiotemporal data, constructing the gait variability, which can be obtained in a convenient, efficient, and inexpensive way in a less time-consuming manner as compared to a laboratory setting [42]. On the most patients with knee OA, they suffer specific knee instability during weight-bearing actions, including lateral thrust, and are required to pay added attention to stability during the stance phase. Our findings show that the lateral thrust and gait variability, especially during the stance phase, were significantly improved using the LWI and was demonstrated by the wearable sensor. In addition, the subjective functional score also improved following the use of LWI. Therefore, these findings indicate the evaluation based on the wearable sensor with clinical tool to detect the ambulatory dysfunction in knee OA, and it also suggests the LWI could apply to patients with knee OA who suffer from gait instability.

This study had several limitations. First, it involved a small number of participants. So, our data did not enough reflect the whole knee OA including different stages and knee alignments. Second, our study included only a single balance parameter which was a self-administered subjective evaluation. Additional parameters could potentially provide more clinically meaningful evidence for a similar study. Third, measurement of long-term improvement was not possible due to the short follow-up period. Fourth, the CV was calculated with 8 steps; more steps could lead to the high reliability of gait variability information [43, 44]. Fifth, we involved the control group by age-matching healthy volunteers without knee OA under different follow-ups. Therefore, we did not obtain the data on nature longitudinal change in knee OA, so it might interfere clearly detection of the effect of LWI in patients with knee OA due to the limiting design of the study. Thus, future research with a larger sample size, analyzing more steps and balance parameters, and robust research design including the comparison with no intervention knee OA group will provide greater insights into the understanding of LWI in patients with knee OA.

## 5. Conclusion

The gait variability based on wearable sensor evaluation and the subjective functional score were improved after wearing the LWI for one-month. LWI can improve ambulatory dysfunction and may be a promising management option for gait instability among knee OA patients.

## Figures and Tables

**Table 1:** Demographic details of participants.

	Knee OA (*n* = 15)	Control (*n* = 13)	*p* value
Age (years)	64.9 ± 9.6	65.0 ± 4.4	0.98
Gender (M : F)	9 : 6	6 : 7	0.46
BMI (kg/m^2^)	25.0 ± 2.8	22.5 ± 1.4	0.008
K/L (II, III, IV)	11, 2, 2		
FTA (°)	176.7 ± 1.8		
Duration of wearing LWI (h/day)	8.1 ± 3.0		

BMI: body mass index, K/L: kellgren-lawrence grade, and FTA: femorotibial angle. Values represent means ± standard deviation.

**Table 2:** The spatiotemporal gait parameters.

	Knee OA baseline	T0	T1	Control	*p* value
Gait speed (m/s)	1.06 ± 0.14	1.08 ± 0.14	1.07 ± 0.14	1.15 ± 0.19	0.448
Stride length (m)	1.12 ± 0.12	1.15 ± 0.12	1.15 ± 0.11	1.21 ± 0.16	0.305
Stride duration (s)	1.07 ± 0.08	1.07 ± 0.08	1.08 ± 0.08	1.06 ± 0.16	0.968
Stance duration (s)	0.56 ± 0.05	0.57 ± 0.04	0.58 ± 0.05	0.57 ± 0.08	0.979
Swing duration (s)	0.50 ± 0.04	0.50 ± 0.04	0.50 ± 0.04	0.49 ± 0.08	0.91

Values represent mean ± standard deviation.

**Table 3:** The clinical evaluation at baseline, T0 and T1 in the knee OA group.

	Baseline	T0	T1
VAS (mm)	36.0 ± 21.5	26.1 ± 18.5^*∗*^	21.0 ± 15.5^*∗*^
KOOS symptom (%)	63.0 ± 15.0		70.0 ± 14.1^*∗*^
KOOS pain (%)	58.8 ± 15.1		65.8 ± 16.4^*∗*^
KOOS function (%)	68.0 ± 16.0		73.2 ± 17.0^*∗*^
KOOS sports (%)	39.0 ± 19.7		47.3 ± 24.1^*∗*^
KOOS QOL (%)	35.6 ± 18.7		46.1 ± 19.7^*∗*^

Values represent mean ± standard deviation. VAS: visual analog scale and KOOS: knee injury osteoarthritis outcome scores. Initial office visit considered as the baseline, wearing insole (T0), and one-month postintervention (T1). ^*∗*^is used to denote significant difference compared with that at baseline (*p* < 0.05).

## Data Availability

The data that support to findings of this study are available from the corresponding authors, upon reasonable request.

## Supplementary Materials

Supplementary Materials This manuscript was confirmed according to the CONSORT checklist.
